# Methylphenidate and Atomoxetine in Pregnancy and Possible Adverse Fetal Outcomes: A Systematic Review and Meta-Analysis

**DOI:** 10.1001/jamanetworkopen.2024.43648

**Published:** 2024-11-06

**Authors:** Ester di Giacomo, Veronica Confalonieri, Fabio Tofani, Massimo Clerici

**Affiliations:** 1 Department of Mental Health and Addiction, Health Care Trust–IRCCS San Gerardo Monza, Monza, Italy; 2 School of Medicine and Surgery, University of Milano-Bicocca, Monza, Italy

## Abstract

**Importance:**

Attention-deficit/hyperactivity disorder (ADHD) is one of the most common neurobehavioral disorders, and it afflicts about 7% of young people. As a consequence, many young women might be pregnant while taking medication for ADHD, but data about safety have not yet been strictly examined.

**Objective:**

To examine adverse effects in offspring of mothers receiving treatment with atomoxetine and methylphenidate during pregnancy.

**Data Sources:**

Electronic databases (PubMed, Embase, and PsycINFO) were searched for articles published through December 31, 2023, with the following search terms: (*atomoxetine* OR *methylphenidate*) AND (*pregnancy*).

**Study Selection:**

Observational studies (eg, cohort studies, case-control studies, case-crossover studies, cross-sectional studies, and registry-based studies) that reported offspring outcomes in pregnancy with atomoxetine and/or methylphenidate and in mothers with ADHD but unexposed to ADHD treatment during pregnancy or from the general population were included. Ten studies of 656 records satisfied criteria.

**Data Extraction and Synthesis:**

Two independent reviewers performed data extraction according to the Preferred Reporting Items for Systematic Reviews and Meta-Analyses guidelines. Meta-analyses were conducted based on reported odds ratios (ORs) and corresponding 95% CIs using a linear random effects model. Each study was inversely weighted by the reported variance of the estimators. Risk of publication bias and analysis of heterogeneity through univariate and multivariate metaregressions were also rated. Data were analyzed from January to March 2024.

**Main Outcomes and Measures:**

Study outcomes included miscarriages and congenital anomalies.

**Results:**

Ten studies involving 16 621 481 pregnant women, 30 830 of them affected by ADHD, were included. Congenital anomalies or miscarriages were not more frequent in offspring of mothers receiving treatment with methylphenidate or atomoxetine during pregnancy compared with unexposed offspring (OR, 1.14; 95% CI, 0.83-1.55; *P* = .41; *I*^2^ = 8% for congenital anomalies; OR, 1.15; 95% CI, 0.82-1.62, *P* = .67; *I*^2^ = 0% for miscarriages) or compared with the general population (OR, 1.19; 95% CI, 0.93-1.53; *P* = .16; *I*^2^ = 74% for congenital anomalies; OR, 0.98; 95% CI, 0.77-1.25; *P* = .40; *I*^2^ = 0% for miscarriage).

**Conclusions and Relevance:**

Evidence from this meta-analysis, which involves a substantial sample of pregnant women with and without ADHD, suggests the maintenance of methylphenidate or atomoxetine during pregnancy is safe, given that congenital anomalies and miscarriages did not appear to significantly increase. Further studies are recommended to support and confirm these findings.

## Key Points

**Question:**

Are atomoxetine and methylphenidate associated with adverse effects on newborns if taken during pregnancy?

**Findings:**

In this systematic review and meta-analysis of 10 studies involving 16.5 million pregnant women, methylphenidate and atomoxetine were not associated with a significant increase in congenital anomalies or miscarriages compared with unexposed pregnancies among women with attention-deficit/hyperactivity disorder (ADHD) or those from the general population.

**Meaning:**

These findings suggest first-line therapy for ADHD should be cautiously continued during pregnancy with a second level of fetal monitoring; further and more detailed studies should be encouraged.

## Teaser

This systematic review and meta-analysis evaluates adverse effects in offspring of mothers taking atomoxetine and methylphenidate during pregnancy.

## Introduction

Attention-deficit/hyperactivity disorder (ADHD) is one of the most common neurobehavioral disorders characterized by hyperactivity, inattention, and impulsivity.^1^ ADHD affects about 3% to 7% of young people worldwide.^2^ About two-thirds of the patients who were diagnosed with ADHD during childhood still have symptoms in adulthood,^3^ with an estimated ADHD prevalence of 3.2% among adult women from the US.^4^

ADHD in adults is characterized by a series of symptoms that, if untreated, may interfere with everyday life and fulfillment of personal well-being and career goals.^5^ The deregulation of executive functions leads to an inability to remain focused on a task for long periods, an inability to prioritize tasks, increased forgetfulness, and difficulty with time management. Emotional dysregulation, low frustration tolerance, and mood lability are other symptoms detectable in untreated adults with ADHD who could therefore face relational and working issues.^6,7^ Moreover, adult women with ADHD seem to be more affected by anxiety, depression, substance use disorder, and self-injury.^8^ Many can experience a multiplicity of struggles in their personal and family relationships as well as at work^9^; in addition, they may feel persistently guilty.^10^

Currently, the pharmacologic treatment for ADHD is the same for both adult women and men. Since most affected women are in their fertile lifespan period, a deep examination of possible adverse effects ADHD treatment may have on fetal well-being is necessary. Moreover, the use of medications to treat ADHD has increased in the last decade, thus involving more fertile women.^11^

Among those medications, the most common are methylphenidate, a molecule belonging to the class of stimulants, and atomoxetine, a selective norepinephrine reuptake inhibitor, which were approved in Italy only about 10 years ago.^12,13^ Both medications have no definitive guidelines or clear indications for their use in pregnancy. In fact, the US Food and Drug Administration (FDA) classifies ADHD drugs as ‘‘pregnancy category C,’’^14,15^ indicating that there are not enough controlled studies in women or that the studies in animals have revealed adverse effects on the fetus. Nonetheless, the number of studies reporting the effects and safety of these drugs for pregnant women is recently increasing. Some studies show a correlation between methylphenidate use during the first trimester of pregnancy and the risk of developing congenital anomalies, in particular cardiac anomalies.^16,17^ However, results are conflicting, and this correlation has not been clearly proved.^18^ Besides, atomoxetine has not been correlated with any neonatal anomalies, but an increased risk of ADHD in the newborn has been suggested, as it has been for methylphenidate.^19^

Based on the evidence, the aim of this study is to attest and quantify possible adverse effects in terms of congenital anomalies or miscarriages in pregnancies of women receiving treatment for ADHD during pregnancy. Furthermore, a secondary goal is to analyze the same outcomes in women with ADHD who do not take specific treatment during pregnancy, thus including those women whose illness is considered less severe and analyzing possible adverse effects of their illness, and not its treatment, on pregnancy.

## Methods

This systematic review and meta-analysis was performed according to the Meta-analysis of Observational Studies in Epidemiology (MOOSE) reporting guidelines. Procedures and study inclusion criteria were defined a priori and registered in PROSPERO (CRD42024403830).

### Data Sources and Search Strategy

A systematic search for articles published in electronic databases (PubMed, Embase, and PsycINFO) through December 31, 2023, was performed with no language or time restrictions. Search phrases combined thesaurus and free-search indexing terms related to ADHD treatment during pregnancy and adverse fetal outcomes using the following search terms: (*atomoxetine* OR *methylphenidate*) AND (*pregnancy*). We contacted corresponding authors of selected studies if additional information was required.

### Eligibility Criteria

We included all observational studies (eg, cohort studies, case-control studies, case-crossover studies, cross-sectional studies, and registry-based studies) comparing pregnant women affected by ADHD and receiving treatment for their disease during pregnancy (hereafter referred to as the exposed group) with women with ADHD but without the assumption of any specific therapy to treat ADHD during pregnancy (hereafter referred to as the unexposed group) or with pregnant women without ADHD from the general population.

We excluded studies without a prespecified comparison group (ADHD unexposed group and pregnant women from the general population) and case reports. We included only studies published in peer-reviewed journals, excluding conference abstracts and dissertations. To reduce the risk of misclassification errors, we included only high-quality studies (according to the Newcastle-Ottawa Scale [NOS] scores; see “Assessment of Study Quality” section), with unequivocal definitions. If data from the same sample were published in multiple works, we retained only the study with more exhaustive information.

### Data Collection Process

Three authors (E.d.G., F.T., and V.C.) preliminarily reviewed titles and abstracts of traced articles. The initial screening was followed by the analysis of full text to check compatibility regarding inclusion and exclusion criteria. Discordance was analyzed and disagreements were resolved by discussion among authors. When reported information was unclear or ambiguous or numerical data were not obtainable by percentages, the relevant corresponding author was contacted for clarification.

### Data Extraction

A standardized form was used to extract data, including information on year of publication, country, setting, characteristics of study participants (sample size and age), gestational characteristics and frequency of adverse events (congenital anomalies and miscarriages), and medications for ADHD. Two authors (E.d.G. and F.T.) conducted data extraction independently; extraction sheets for each study were cross-checked for consistency, and any differences were resolved by discussion among the coauthors.

### Assessment of Study Quality

Three authors (E.d.G, V. C., and M.C.) used the NOS^20^ to independently rate the quality of the included studies. Any discrepancy was resolved by consensus. The NOS is a tool used for assessing the quality of nonrandomized studies included in systematic reviews and/or meta-analyses. Using the tool, each study is judged on 8 items categorized into 3 groups: the selection of the study groups, the comparability of the groups, and the ascertainment of either the exposure or outcome of interest for case-control or cohort studies.

### Statistical Analysis

We performed a meta-analysis of the ORs of miscarriages and congenital anomalies among offspring of the exposed group compared with the pregnant unexposed group, and we generated pooled ORs with 95% CIs using linear inverse variance models with random effects. Moreover, an in-depth analysis about the same comparison among the unexposed group in comparison with the general population is performed, thus investigating a possible association between ADHD itself in possible congenital anomalies or miscarriages rather than the medications used to treat it. Our goal was to improve comprehension about these issues in pregnant women affected by ADHD and results generalizability.

Results were summarized using conventional forest plots. Standard χ^2^ tests and the *I*^2^ statistic (ie, the percentage of variability in prevalence estimates attributable to heterogeneity rather than sampling error or chance, with values 75% or greater indicating high heterogeneity) were used to assess between-study heterogeneity. To test for publication bias, we performed funnel plot analysis and the Egger test, performing 6 separate Egger tests. The Egger test quantifies bias captured in the funnel plot analysis with linear regression using the value of effect sizes and their precision (SE) and assumes that the quality of study conduct is independent of study size. If analyses showed a significant risk of publication bias, we would use the trim and fill method to estimate the number of missing studies and the adjusted effect size. Meta-regression analysis was performed to examine sources of between-study heterogeneity if of a high level (*I*^2^ > 75%) on a range of study prespecified characteristics (ie, sample size, age, and country). All analyses were performed using the meta and metaphor packages in R, version 3.2.3 (R Project for Statistical Computing). Statistical tests were 2-sided and used a significance threshold of *P* < .05. Data were analyzed from January to March 2024.

## Results

### Study Characteristics

**Figure 1.**
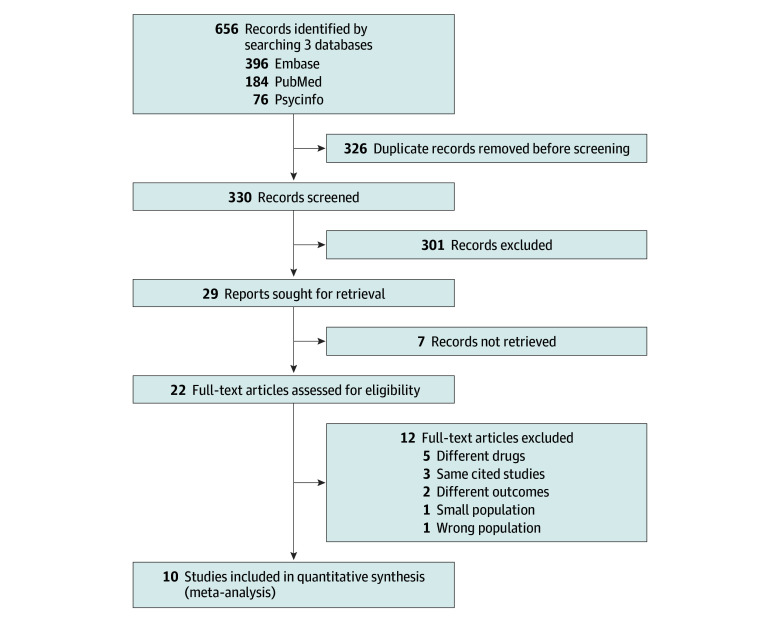
PRISMA Flow Chart

Ten studies^5,11,16,17,18,22,23,24,25,26^ were included in the quantitative analysis (Figure 1), examining 16 621 481 pregnant women, 30 830 of them affected by ADHD. The studies were conducted in 6 countries (Denmark, Sweden, Norway, Iceland, US, Israel, and the Netherlands). All studies were published after 2014, with 5 of them published after 2020 (see study characteristics in Table). The studies varied in quality, with NOS ratings ranging from 7 to 9, thus indicating good or high-quality studies (see eTable 1 in Supplement 1).

**Table.** Study Characteristics

Source name, year (country)	Drug	Events, No.											
		Drug exposed pregnancy				No-drug exposed pregnancy				General population			
		Events in ADHD			ADHD	Events in ADHD			ADHD	Events in pregnancy			Pregnancy
		Miscarriages	Congenital abnormalities			Miscarriages	Congenital abnormalities			Miscarriages	Congenital abnormalities		
			Cardiac anomaly	Congenital anomaly			Cardiac anomaly	Congenital anomaly			Cardiac anomaly	Congenital anomaly	
Bro et al,^22^ 2015 (Denmark)	Methylphenidate and atomoxetine	18	NA	2	186	26	NA	7	275	114 672	NA	39 557	989 471
Bröms et al,^26^ 2023 (Northern Europe and US)	Atomoxetine	NA	20	42	990	NA	NA	NA	NA	NA	53 174	152 052	4 238 544
Damer et al,^23^ 2021 (the Netherlands)	Methylphenidate	NA	NA	1	26	NA	NA	3	82	NA	NA	NA	NA-
Damkier and Broe,^24^ 2020 (Denmark)	Methylphenidate and atomoxetine	NA	NA	43	963	NA	NA	NA	NA	NA	NA	32 466	828 644
Hærvig et al,^25^ 2014 (Denmark)	Methylphenidate and atomoxetine												
	Congenital anomalies	NA	NA	3	505	NA	NA	NA	NA	NA	NA	41 241	1 054 494
	Miscarriages	71	NA	NA	681	48	NA	NA	505	114 389	NA	NA	1 054 494
Huybrechts et al,^17^ 2018 (US)	Methylphenidate	NA	39	95	2072	NA	NA	NA	NA	NA	22 910	62 966	1 797 938
Kolding et al,^16^ 2021 (Denmark)	Methylphenidate	NA	31	24	473	NA	NA	NA	NA	NA	10 182	16 857	363 219
Ornoy and Koren,^5^ 2021 (Israel)	Methylphenidate	NA	NA	10	309	NA	NA	13	358	NA	NA	NA	NA
Nörby et al,^11^ 2017 (Sweden)	Methylphenidate and atomoxetine	8	NA	48	1591	38	NA	205	9475	4268	NA	20 736	953 668
Pottegard et al,^18^ 2014 (Denmark)	Methylphenidate	NA	3	7	222	NA	32	86	2201	NA	NA	NA	NA

Abbreviations: ADHD, attention-deficit/hyperactivity disorder; NA, not applicable.

### Analysis of ADHD Drug Exposed vs Unexposed Pregnancies

**Figure 2.**
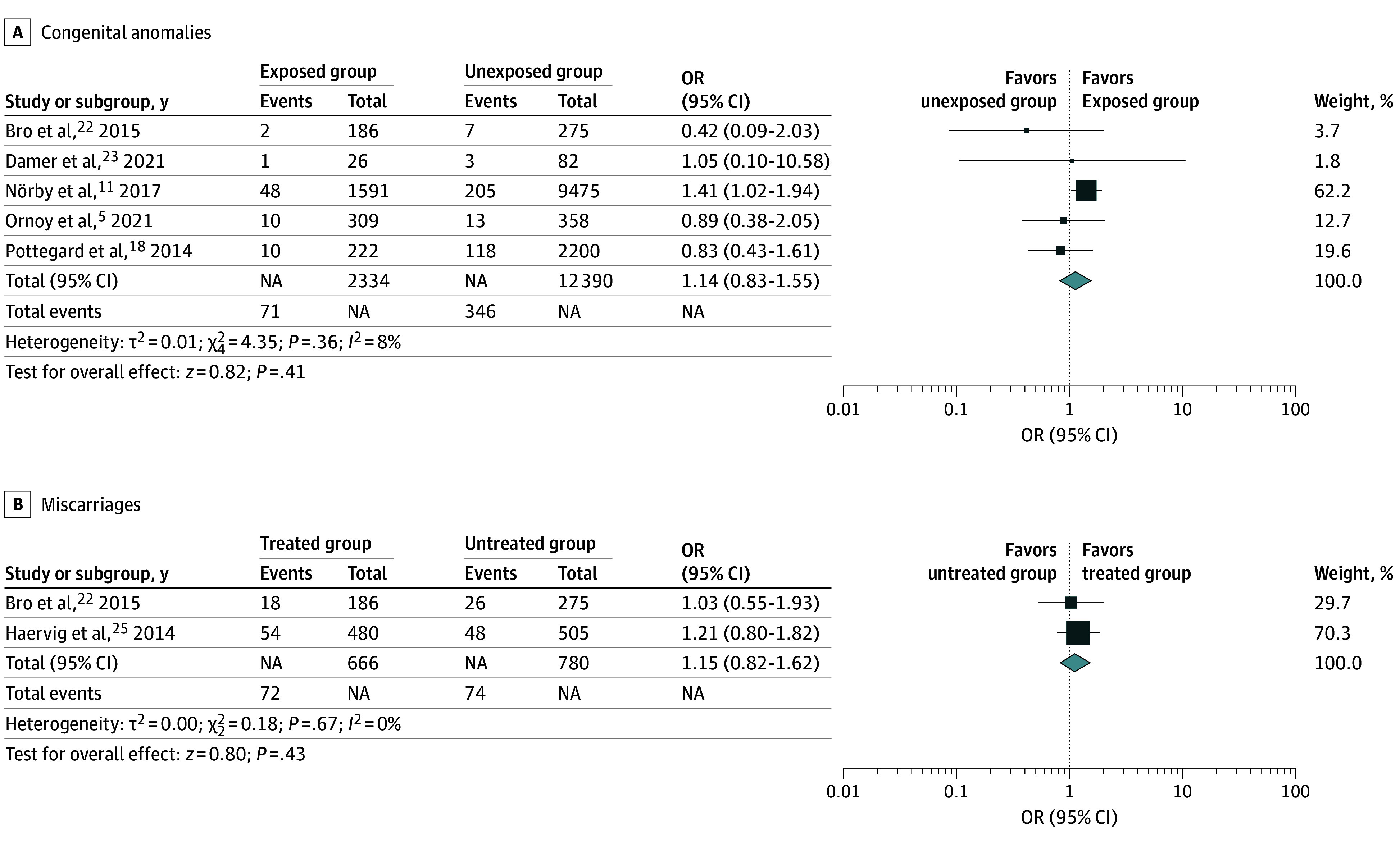
Exposed Group vs Unexposed Group Different box sizes indicate study weight. NA indicates not applicable; OR, odds ratio.

Congenital anomalies were not more frequent in offspring of the exposed group compared with offspring of the unexposed group (OR, 1.14; 95% CI, 0.83-1.55; *P* = .41; *I*^2^ = 8%) (Figure 2). Miscarriages also were not more frequent in offspring of the exposed group compared with offspring of the unexposed group (OR, 1.15; 95% CI, 0.82-1.62, *P* = .67, *I*^2^ = 0%).

### Analysis of ADHD Drug–Exposed Pregnancy Compared With the General Population

**Figure 3.**
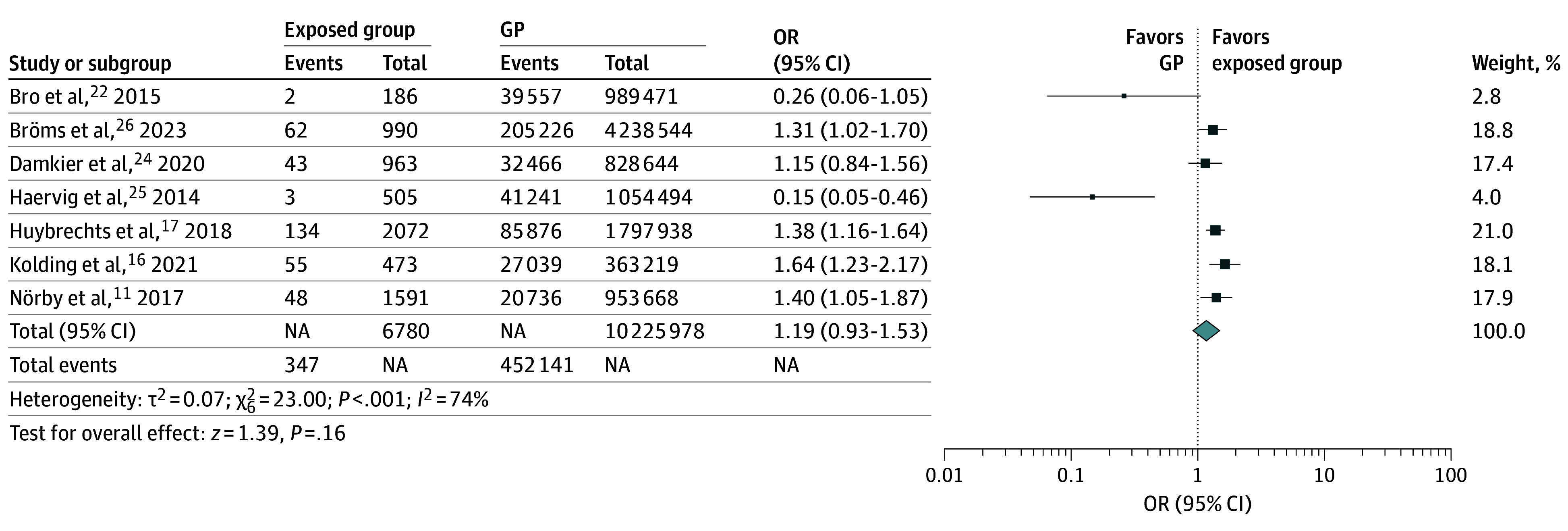
Exposed Group vs General Population (GP) Forest plot of comparison: congenital anomalies in exposed group vs general population. Different box sizes indicate study weight. NA indicates not applicable; OR, odds ratio.

Congenital anomalies were not more frequent in offspring of the exposed group compared with the general population (OR, 1.19; 95% CI, 0.93-1.53; *P* = .16; *I*^2^ = 74%) (Figure 3). There was no difference in miscarriage frequency in offspring of the exposed group compared with the general population (OR, 0.98; 95% CI, 0.77-1.25; *P* = .40; *I*^2^ = 0%).

### In-Depth Analysis of ADHD-Unexposed Pregnancies vs General Population

**Figure 4.**
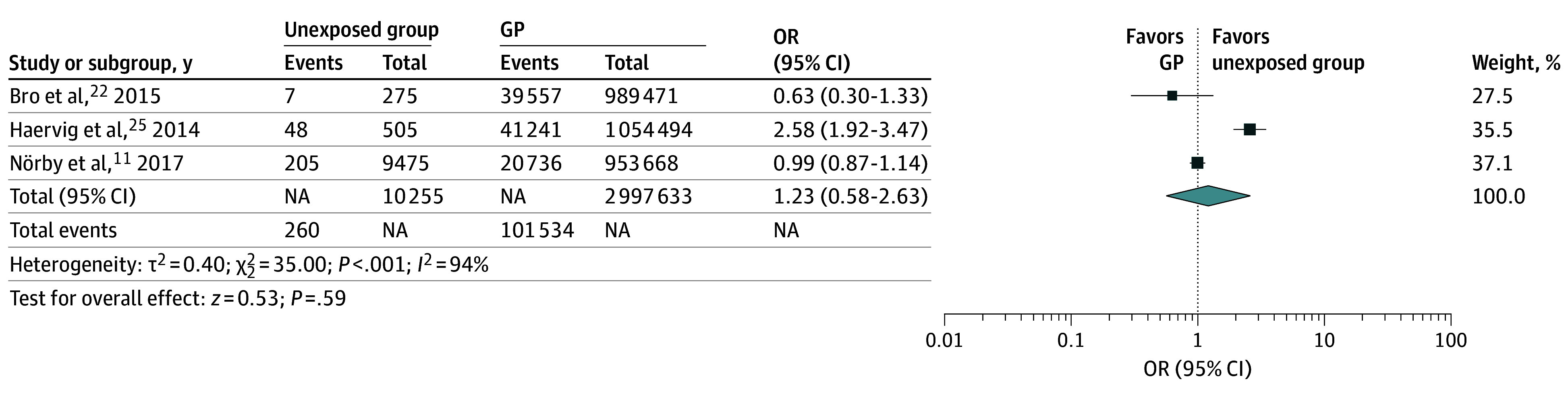
Unexposed vs General Population (GP) Forest plot of comparison: congenital anomalies in unexposed group vs general population. Different box sizes indicate study weight. NA indicates not applicable; OR, odds ratio.

There were no statistical differences in frequency of congenital anomalies in the offspring of the unexposed group compared with the general population (OR, 1.23; 95% CI, 0.58-2.63; *P* = .59; *I*^2^ = 94%). There were also no statistical differences in the frequency of miscarriages (OR, 0.84; 95% CI, 0.66-1.07, *P* = .75; *I*^2^ = 0%) (Figure 4).

### Publication Bias

The risk for publication bias existed in the exposed group vs the general population for congenital anomalies (Egger test, −3.32; 95% CI, −5.46 to −1.17; *P* = .01). The trim and fill method trimmed 3 studies to the right of the mean (OR, 0.78; 95% CI, 0.90 to 1.85; Cochran *Q* = 40.768). The remaining comparisons did not show significant risks for publication bias (exposed group vs unexposed group for congenital anomalies: Egger test, −1.26; 95% CI, −3.75 to 1.24; *P* = .30; unexposed group vs the general population for congenital anomalies: Egger test −2.26; 95% CI, −48.66 to 44.14; *P* = .65; exposed group vs unexposed group for miscarriages: Egger test −1.79; 95% CI, −4.84 to 12.24; *P* = .16; unexposed group vs the general population for miscarriages: Egger test −1.45; 95% CI, −10.12 to 11.11; *P* = .39; exposed group vs the general population for miscarriages: Egger test −2.50; 95% CI, −51.04 to 58.09; *P* = .41).

### Sources of Heterogeneity

Heterogeneity was detected between studies focused on congenital anomalies in exposed group pregnancies vs the general population and among studies on congenital anomalies in the unexposed group vs the general population. Univariate and multivariate metaregressions were performed, considering ORs as outcomes and weighted with study size, according to the DerSimonian-Laird method.^21^ The following variables were used: country and year of the study. Those variables were not significantly associated with congenital anomalies in the exposed group vs the general population, but they were both significant in congenital anomalies in unexposed vs the general population (see eTable 2 in Supplement 1).

## Discussion

To our knowledge, this is the first extensive meta-analysis involving more than 16 million pregnant women that examines possible adverse outcomes in the offspring of mothers with ADHD who take specific drug therapies for ADHD (methylphenidate and atomoxetine) during pregnancy compared with mothers with the same pathology but without prescription during pregnancy and the general population. The sample of pregnant women with ADHD was considerable, with about 30 000 participants, thus contributing to the generalizability of results. In addition, the analysis of pregnant women with ADHD who were not taking medications compared with those from the general population, which contributes to the inclusion of patients with ADHD whose illness might be less severe and shows a possible contribution (or exclusion) of ADHD and not its treatment in determining congenital anomalies or abortions also contributed to the generalizability of results. Li and colleagues^27^ led a large and important qualitative review in 2020, analyzing several possible treatments for ADHD and including 8 studies in their qualitative analysis with the conclusion that “there was no convincing evidence to indicate that prenatal exposure to ADHD medication results in clinically significant adverse effects.”^27^ The present meta-analysis aimed at quantifying the possible adverse congenital outcomes of the leading molecules prescribed for ADHD to support the most precise recommendation.

Women affected by ADHD who were receiving treatment with atomoxetine or methylphenidate during pregnancy did not show a significantly higher frequency of congenital anomalies or miscarriages compared both with those without ADHD-treatment intake during pregnancy and with the general population. Interestingly, the in-depth analysis of mothers with unexposed ADHD during pregnancy suggests that the frequencies of congenital anomalies or miscarriages are not significantly different compared with the general population, possibly supporting a low association between ADHD genetics and those issues.

The present research is an extensive analysis of the literature aimed at exploring actual knowledge about teratogenic and adverse implications of the most common ADHD treatment intake during pregnancy. The sample analyzed is very large, and generalizability is high because of the substantial population and comparison of pregnant women with unexposed ADHD with those from the general population. ADHD is a common disorder in the population with an onset during childhood and a lifelong continuity. Accordingly, many women might desire a pregnancy while receiving treatment for ADHD but may have worries due to the lack of precise scientific knowledge.

The present meta-analysis highlights a substantial absence of adverse effects in terms of congenital anomalies or miscarriages. Furthermore, no significant difference has been detected between women with unexposed ADHD during pregnancy and the general population, highlighting a plausible absence of genetic influence of ADHD on offspring’s congenital anomalies or miscarriages.

### Limitations

Even though the analysis is based on a substantial sample, some limitations should be stressed. First of all, the definition of congenital anomaly is not specific and may range importantly. Above all, the term does not imply the level of seriousness or any specificity of the anomaly. Second, it might be supposed that women who have to maintain their treatment during pregnancy could have a more severe form of ADHD, but this is only an assumption since the severity of ADHD is not specified in many studies. To avoid any issue regarding generalizability of results, we specifically analyzed pregnant women affected by ADHD who were not receiving treatment during pregnancy compared with the general population.

## Conclusions

This is, to our knowledge, the first extensive meta-analysis of possible adverse effects in offspring due to methylphenidate and atomoxetine intake during pregnancy. It also analyzed those effects within patients affected by the same disorder (receiving treatment or unexposed) and in comparison with the general population. Methylphenidate and atomoxetine intake during pregnancy were not associated with higher frequencies of congenital anomalies or miscarriages in this meta-analysis. Even if results are prudentially comforting, further studies are needed to support pregnant women with ADHD in facing a comfortable pregnancy, possibly maintaining the well-being they receive from treatments for their disorder, and avoiding relapses.

## Notes

Supplement 1. **eTable 1. **Quality Assessment by Newcastle-Ottawa Scale **eTable 2. **Metaregression

Supplement 2.
Data Sharing Statement
