# Error in Abstract, Results, and Figure

**DOI:** 10.1001/jamanetworkopen.2025.10141

**Published:** 2025-04-08

In the Original Investigation titled “Methylphenidate and Atomoxetine in Pregnancy and Possible Adverse Fetal Outcomes: A Systematic Review and Meta-Analysis,”^1^ published November 6, 2024, there were errors in the Abstract, Results, and Figure. Events from a cited study were misclassified. After reanalysis, effect sizes and 95% CIs were adjusted, but overall findings did not change. The authors have explained what happened in a Comment.^2^ This article has been corrected.^1^
