# Supplementary material for: Methylphenidate and Atomoxetine in Pregnancy and Possible Adverse Fetal Outcomes: A Systematic Review and Meta-Analysis
Source: JAMA Netw Open. 2024 Nov 6;7(11):e2443648. doi: 10.1001/jamanetworkopen.2024.43648 (PMC11541644; doi:10.1001/jamanetworkopen.2024.43648)
Supplement: Supplement 1. — eTable 1. Quality Assessment by Newcastle-Ottawa Scale eTable 2. Metaregression [file jamanetwopen-e2443648-s001.pdf]

# Supplemental Online Content

di Giacomo E, Confalonieri V, Tofani F, Clerici M. Methylphenidate and atomoxetine in pregnancy and possible adverse fetal outcomes: a systematic review and meta-analysis. *JAMA Netw Open*. 2024;7(11):e2443648. doi:10.1001/jamanetworkopen.2024.43648

**eTable 1.** Quality Assessment by Newcastle-Ottawa Scale
**eTable 2.** Metaregression

This supplemental material has been provided by the authors to give readers additional information about their work.

© 2024 di Giacomo E et al. *JAMA Network Open.*

| Study | Selection | Comparability | Outcome | Total |
|---|---|---|---|---|
| Bro et al, 2015 | **** | ** | *** | 9 |
| Bröms et al., 2023 | **** | ** | *** | 9 |
| Damer et al., 2021 | **** | * | *** | 8 |
| Damkier & Broe , 2020 | **** | ** | *** | 9 |
| Hærvig et al., 2014 | **** | * | *** | 8 |
| Huybrechts et al., 2018 | **** | * | *** | 8 |
| Kolding et al., 2021 | **** | * | *** | 8 |
| Ornoy & Koren, 2021 | **** | * | *** | 8 |
| Nörby et al., 2017 | **** | * | *** | 8 |
| Pottegard et al., 2014 | **** | * | *** | 7 |

**eTable 1.** Quality Assessment by Newcastle-Ottawa Scale

**eTable 2.** Metaregression

| Comparison | Heterogenity source | $\chi^2$ | $R^2$ | p |
|---|---|---|---|---|
| Congenital malformations in "$ADHD_{MPH/ATX}$"-vs GP | Country | 10.469 | 0 | .063 |
| | Year of publication | 8.504 | 16.9 | .131 |
| | Country + Year of pubblication | 5.794 | 46.4 | .215 |
| Congenital malformations in unexposed vs GP | Country | 104.012 | 0 | <.001 |
| | Year of publication | 76.335 | 0 | <.001 |
| | Country + Year of publication | 76.335 | 0 | <.001 |

© 2024 di Giacomo E et al. *JAMA Network Open.*
