# Supplementary material for: Methylphenidate and Atomoxetine in Pregnancy and Possible Adverse Fetal Outcomes: A Systematic Review and Meta-Analysis
Source: JAMA Netw Open. 2024 Nov 6;7(11):e2443648. doi: 10.1001/jamanetworkopen.2024.43648 (PMC11541644; doi:10.1001/jamanetworkopen.2024.43648)
Supplement: Supplement 2. — Data Sharing Statement [file jamanetwopen-e2443648-s002.pdf]

Data Sharing Statement

di Giacomo. Methylphenidate and Atomoxetine in Pregnancy and Possible Adverse Fetal Outcomes. *JAMA Netw Open*. Published November 06, 2024.
doi:10.1001/jamanetworkopen.2024.43648

Data
Data available: Yes
Data types: Data dictionary
How to access data: Prospero
When available: With publication

Supporting Documents
Document types: Other (please specify)
Additional Information: protocol
How to access documents: PRISMA checklist
When available: With publication

Additional Information
Who can access the data: anyone requesting the data
Types of analyses: for any purpose
Mechanisms of data availability: approval of a proposal
